# Spargel/dPGC-1 influences cell growth through the E2F1-mediated endocycle pathway

**DOI:** 10.1093/g3journal/jkag180

**Published:** 2026-07-06

**Authors:** Md Shah Jalal, Atanu Duttaroy

**Affiliations:** Biology Department, Howard University, Washington, DC 20059, United States; Department of Molecular Biophysics and Biochemistry,Yale University, New Haven, CT 06510, United States; Biology Department, Howard University, Washington, DC 20059, United States

**Keywords:** *Drosophila*, Spargel, dPGC-1, PGC-1, endocycle, growth, E2F1

## Abstract

The endocycle is a specialized variant of the eukaryotic cell cycle program observed in various tissues across diverse organisms ranging from insects to mammals. The endocycle promotes cellular growth by oscillating between the synthesis (S) and gap (G) phases, completely bypassing mitosis (M phase). E2F1 serves as a master regulator of the endocycle in *Drosophila* salivary glands, whereas the target of rapamycin (TOR) signaling pathway controls the levels of the E2F1 protein post-transcriptionally. Cellular growth in tissues that undergo the endocycle is also dependent on nutrient availability. *Drosophila* Spargel (dPGC-1) is orthologous to the PGC-1 family of transcriptional coactivators in vertebrates. In flies, Spargel influences cell growth through the insulin signaling pathway via TOR. However, the mechanisms by which Spargel regulates endocycle-mediated growth have yet to be established. Here, we report an essential role for Spargel in the *Drosophila* larval salivary gland that influences E2F1-mediated cellular growth. To elucidate the role of Spargel in cellular growth, we performed flippase (FLP)/FLP recombination target (FRT)-mediated mitotic clonal analysis in the salivary gland. The clonal analysis revealed that a cell-specific, complete loss of Spargel results in smaller nuclei with reduced DNA content due to inadequate DNA replication. Furthermore, the selective absence of Spargel abrogates the expression of a key DNA replication factor, E2F1, which promotes the G1 → S transition in the endocycle. Thus, mechanistically, Spargel plays a key role in cell growth by positively influencing the endocycle process through the E2F1 pathway.

## Introduction

During development, growth determines body size, which ultimately depends on the number and size of cells in the adult organism (Conlon and Raff 1999; Tennessen and Thummel 2011). *Drosophila* is a recognized model for studying growth because its juvenile larval stage undergoes dramatic changes, leading to a remarkable ∼200-fold increase in body mass (Church and Robertson 1966; Tennessen and Thummel 2011). The initial growth of *Drosophila* embryos is mitotic, whereas postembryonic growth relies entirely on increases in cell size in larval endoreplicating tissues (Church and Robertson 1966; Gupta et al. 2013). *Drosophila* larval tissues shift from mitosis to a different cell cycle program called the endocycle, which includes only the synthesis (S) and gap (G) phases and omits cytokinesis (Edgar et al. 2014).

*Drosophila* larvae possess a pair of salivary glands, each comprising approximately 100 cells (Andrew et al. 2000). Once cells are committed to forming the salivary glands, no cell division or cell death occurs within these glands until histolysis (Andrew et al. 2000). Throughout larval development, the salivary gland cells increase in size through endocycle (Shu et al. 2018; Loganathan et al. 2021). The endocycle leads to polyploid cells with multiple copies of their genome, thereby driving growth (Shu et al. 2018). The transcription factor E2F1 plays a central role in regulating the endocycle in *Drosophila* larval salivary gland (Kim et al. 2021), and the three upstream effectors of E2F1, namely Myc, the target of rapamycin (TOR), and receptor tyrosine kinases (RTK), regulate the endoreplication pace (Edgar et al. 2014).

The peroxisome proliferator-activated receptor γ coactivator 1 (PGC-1) group of transcriptional coactivators serves as a master regulator of mitochondrial biogenesis and energy metabolism in mammals (Andersson and Scarpulla 2001; Lin et al. 2002; Lin et al. 2003). The discovery of *Drosophila* Spargel/dPGC-1 as the ancestral ortholog of mammalian PGC-1 family (Tiefenböck et al. 2010; Roy et al. 2023) led us to explore the biology of this essential transcriptional coactivator. Previous genetic and cell-biological studies have documented the indispensable role of Spargel/dPGC-1 in germ cell growth in female fertility (Basar et al. 2019) and in eggshell biogenesis (Jalal and Duttaroy 2024). However, the molecular mechanism of how Spargel regulates growth in *Drosophila* remains poorly understood. To elucidate the role of Spargel in growth, we chose *Drosophila* salivary glands, which undergo approximately 10 rounds of asynchronous endocycles (Hammond and Laird 1985), thereby offering a valuable opportunity to comprehensively investigate endocycles using the extensive array of available genetic tools. In this report, we show that Spargel/dPGC-1 is localized to the cytoplasmic compartment of the salivary gland and positively regulates the endocycle during the postembryonic development of *Drosophila*. Thus, Spargel is involved in cellular and, consequently, organismal growth through endocycle.

## Methods

### *Drosophila* strains and genetics

All the fly stocks were raised on standard cornmeal agar medium at a constant temperature (23 °C). The following *Drosophila melanogaster* stocks were obtained from BDSC: *w^1118^ (w[1118]), y[1]; P{y[ + mDint2] w[BR.E.BR] = SUPor-P}srl[KG08646] ry[506]/TM3, Sb[1] Ser[1]* (BDSC #14965), w[*]; P{w[ + mC] = matalpha4-GAL-VP16}V37 (BDSC #7063), *y[1]*  w[*];  *P{w[+mW.hs]=GawB}AB1]* (BDSC #1824), y[1] w[*];  *P{w[+mC]=tubP-GAL4}LL7/TM3, Sb[1] Ser[1]* (BDSC #5138), y[1] sc[*] v[1] sev[21]; P{y[ + t7.7] v[ + t1.8] = TRiP.HMS00857}attP2 (*UAS-srl^RNAi^*, BDSC #33914).

### Generation of *srl^null^* cell clones

*spargel* loss-of-function (*srl^null^*) clones were generated in the larval tissues using mitotic recombination. A CRISPR-mediated new FRT insertion at FRT81F6 was generated first because the canonical FRT82B6 insertion is near the *spargel* locus, which impedes mitotic recombination (Roy et al. 2023). ry,hsflp,y,w*; ;FRT81F6,srl^del^/TM3,ry, Sb males were crossed to w*; P{w[ + mC] = Act5C-GAL4}25FO1/CyO; FRT81F6, UAS-GFP,w+/TM3, Sb virgin females. The 4- to 5-h F1 embryos from the above cross were collected on grape agar plates and heat-shocked once for 1.5 h at 37 °C. Heat-shocked samples were raised in an incubator (23 °C) for larval growth. Then, the larval tissues, including the salivary glands, were dissected from the desired larval stage (wandering third instar) for further analysis. *srl^null/null^* clones in the salivary gland cells were identified by the absence of a GFP marker.

### Immunostaining and antibodies

The salivary glands were dissected in Grace's Insect Medium (Cat. No. 11605–094, Life Technologies), fixed in 1× phosphate-buffered solution (PBS) with 4% paraformaldehyde (Cat. No. 18505, Ted Pella) for 20 min at room temperature [note: for anti-E2F1, tissues were fixed (30 min), washed and blocked at 4 °C]. Tissues were then washed three times in 1× PBS with 0.3% Triton-X-100 (PBST) and blocked in a 1% BSA in PBST (PBTB) for 1 h at room temperature. The tissues were incubated in PBTB with an appropriate primary antibody overnight at 4 °C, then washed three times in PBST at room temperature. The secondary antibody incubation was carried out for 1 h at room temperature in the dark. Then, the tissues were washed three times in PBTB and mounted in VECTASHIELD antifade mounting medium with 4′, 6-diamidino-2-phenylindol (DAPI) (Cat. No. H-1200-10; Vector Laboratories) for imaging. The following primary and secondary antibodies were used in this study: mouse anti-Spargel (1:300, 7A10), rabbit anti-E2F1 (1:500, Maxim Frolov Lab), rhodamine Red-X goat antimouse (Molecular Probes, Life Technologies), and rhodamine Red-X goat antirabbit (Molecular Probes, Life Technologies).

### 5-Ethynyl-2′-deoxyuridine (EdU) incorporation

A Click-iT EdU Alexa Fluor 555 Imaging Kit (Cat. No. C10338, Life Technologies) was employed to incorporate 5-ethynyl-2′-deoxyuridine (EdU) into the *Drosophila* salivary gland following the manufacturer's protocol. In brief, salivary glands from wandering third-instar larvae were dissected and incubated for 1 h at room temperature in Grace's medium (Life Technologies) with 10 μM EdU in a Petri dish. The EdU solution was then removed, and the glands were rinsed twice for 3 min each in Grace's medium to eliminate unincorporated label. The samples were fixed and blocked as described previously. Subsequently, they were washed in PBTB for 20 min, incubated with an EdU reaction cocktail for 30 min, and then washed for 2 h before mounting.

### Microscopy

The immunostained and EdU-labeled samples were imaged with a Nikon Ti-E-PFS inverted confocal microscope equipped with a Yokogawa CSU-X1 spinning disk using the 40× (oil), 20×, and 10× Plan Apo λ objective lenses. The NIS Elements Ar imaging software was used to adjust the gamma, brightness, and contrast. Adult flies and the DAPI-stained salivary glands were photographed and acquired with a Zeiss Axioskop 2 plus microscope in brightfield mode, equipped with a PhotoFluor LM-75 light source using a 5× or 10× Zeiss Fluar objective lens.

### Quantification of fluorescence intensity

To quantify fluorescent intensity, maximum-intensity Z-projection images were used for DAPI, EdU, and E2F1. Circular regions of interest (ROIs) were drawn in NIS-Elements software around the nuclei, and the mean intensity within each ROI was measured. Intensity profiles were measured without background subtraction and exported to a multicolumn.xlsx file for each ROI.

### Statistical analysis

A minimum of three independent samples were dissected, fixed, and stained under identical conditions. Image acquisition settings remained consistent across all images used for quantification, ensuring uniformity in intensity measurements. Quantification and statistical analysis were conducted with at least three independent replicates collected under the same conditions. Students' *t*-tests were used to determine statistical significance and compare the two data groups. Statistical software, including GraphPad Prism 9 and Microsoft Excel, was used to analyze and generate bar charts. The following *P*-values were evaluated for statistically significant differences: *P ≤ 0.05, **P ≤ 0.01, ***P ≤ 0.001, ****P ≤ 0.0001.

## Results

### Spargel expression pattern in developing somatic tissues like muscle, gut, and salivary gland

**Fig. 1.**
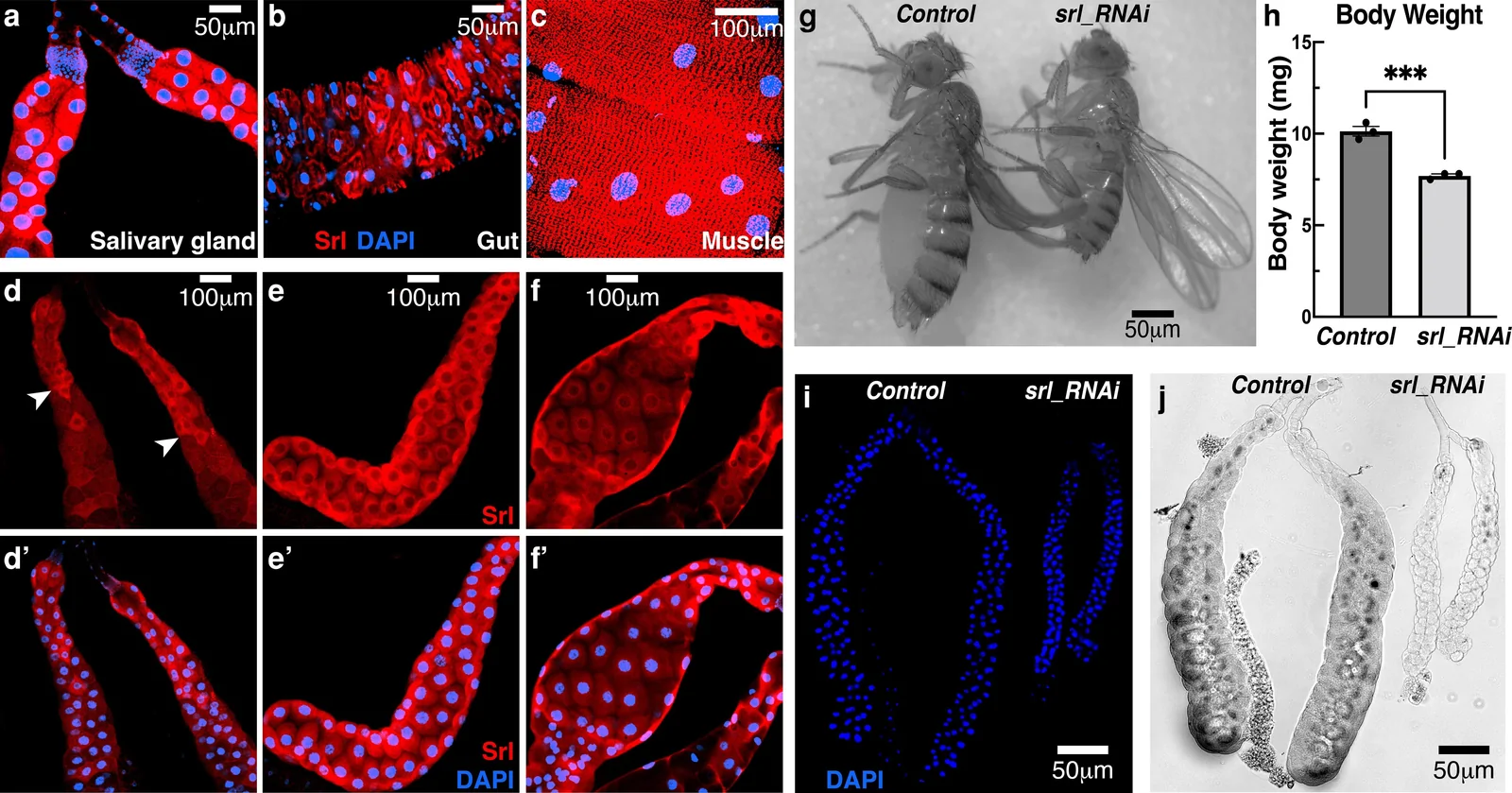
Spargel protein expression profile in larval somatic tissues. Immunostaining with the Spargel-specific monoclonal antibody (clone 7A10) reveals cytoplasmic localization of Spargel in all examined larval somatic tissues: a) salivary gland, b) gut, and c) muscle tissues obtained from *w[1118]* wandering third instar larva. Nuclei were counterstained with DAPI. Scale bars are defined as: 50 µm for a) and b) and 100 µm for c). d–f) The distribution and expression pattern of Spargel in the salivary gland during the later stages of larval development. In the third instar larvae, Spargel expression appears in the proximal region (white arrowheads) of the glands d). In contrast, in prepupal and pupal stages, a notable extension of cytoplasmic Spargel expression spreads from the proximal to the distal region of the gland e), which persists until gland histolysis f). d′–f′) Merged panels of the salivary glands, where nuclei were stained with DAPI. The scale bars 100 µm. Ubiquitous knockdown of *spargel* (*tubulin_gal4 > UAS-srl-RNAi*) causes reduced body size g) and produces flies with low body weight h). A comparison of average adult body weight between *control* and *srl-RNAi* flies confirms a significant reduction in body weight in *srl-RNAi* flies. Body weight was measured in adult flies. Salivary gland-specific (*AB1_gal4 > UAS-srl-RNAi*) downregulation of *spargel* results in thinner and smaller glands; DAPI-stained salivary glands i) and the same glands in brightfield j). The scale bars 50 µm.

Late-stage embryonic lethality of *spargel* (*srl*) null embryos specifies a zygotic requirement for this transcriptional coregulator during *Drosophila* development (Roy et al. 2023). Earlier studies documented that Spargel is highly expressed in the nuclei of ovarian germ and follicle cells in adult females (Basar et al. 2019). In contrast, Spargel has also been reported to be expressed in the cytoplasm of fat body cells (Tiefenböck et al. 2010). Using Spargel monoclonal antibody (Basar et al. 2019), we performed a detailed survey of Spargel expression in major larval tissues, including salivary gland, gut, and muscles (Fig. 1a and c). The presence of Spargel in the cytoplasm of these larval tissues supports previous findings that Spargel is localized within cytoplasmic compartments in most somatic tissues (Tiefenböck et al. 2010; Mukherjee et al. 2013). However, more dynamic spatiotemporal changes in cytoplasmic Spargel expression pattern were observed in the larval salivary gland during larval to pupal transition. In wandering third instar larvae, Spargel is expressed predominantly in the proximal region of the glands (Fig. 1d and d′). No Spargel expression is evident in the distal region of the gland during this time (Fig. 1d and d′). However, in prepupal and pupal stages, the cytoplasmic expression pattern of Spargel expands from the proximal to the distal region, and eventually it is expressed throughout the glands (Fig. 1, e, f, e′, and f′). The above observation raises questions about the biological relevance of Spargel being localized to two distinct subcellular compartments in somatic and germ cells.

### Ubiquitous and tissue-specific loss of spargel displays a growth defect

To investigate the role of Spargel in the developing larval tissues, we performed both ubiquitous and salivary gland-specific knockdowns of *spargel* using *tubulin_gal4* and *AB1_gal4* drivers, respectively. In each case, *spargel* knockdown results in somatic growth arrest. The global knockdown of *spargel* produces smaller body size and significantly underweight flies (Fig. 1g and h), whereas the salivary gland-specific knockdown of *spargel* results in rudimentary glands (Fig. 1i and j). Downregulation of *spargel* in the salivary gland mimics the *spargel* knockdown phenotype in ovaries (Basar et al. 2019). Therefore, the global and tissue-specific suppression of *spargel* indicates a correlation between *spargel* and growth, as the downregulation of *spargel* leads to reduced growth globally and in a tissue-specific manner. These observations indicate a strong link between Spargel and growth. To address the correlation between growth and Spargel, we investigated the cellular requirements of Spargel using a widely employed mitotic clonal technique in *Drosophila*.

### Cell-specific loss of *Spargel* supports its role in endocycle-mediated cell growth

**Fig. 2.**
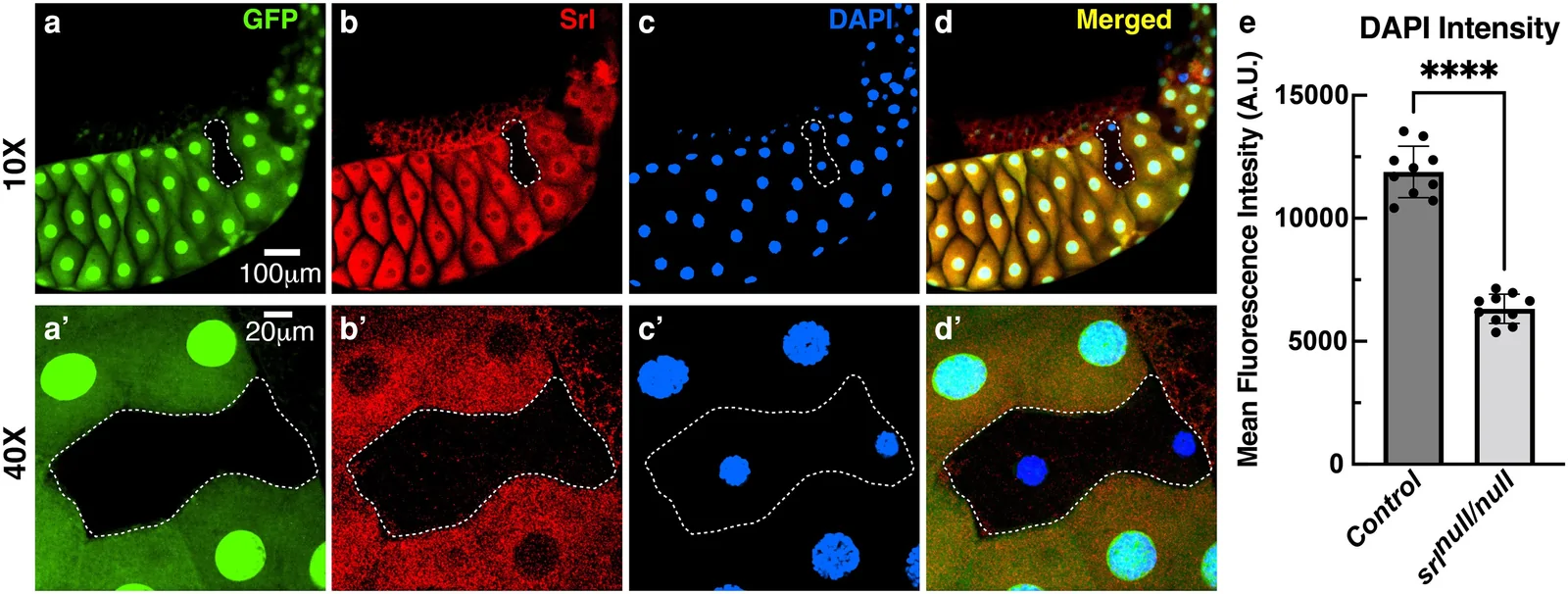
Cell-autonomous loss of *spargel* function in the salivary gland leads to smaller nuclear size. a–d and a′–d′) The same tissue at 10× and 40× magnifications, respectively. Both panels show that nuclear size reduction is evident at both tissue-wide and cellular levels. a–a′) *srl^null^* clones are GFP negative, whereas GFP-positive cells are *control* cells. b–b′) GFP-negative cells are also negative for Spargel expression (immunostained with anti-Spargel). c–c′) DAPI-stained *srl^null^* nuclei (white dotted box) are much smaller in size compared to the adjacent control cell nuclei. d-d′) Merged images of panels a-c and a'-c′. e) Quantification of DAPI intensities in the nuclei of *control* cells compared to the *srl^null^* clone cells. The number of dots represents the number of nuclei counted in each group. The error bars represent SEM, and the *P* value is <0.0001. *n* = 10. White lines across all panels indicate the clone boundary. Scale bar, 100 µm (top panel) and 20 µm (bottom panel).

The mitotic clonal technique is a widely used genetic method for generating mosaic tissues or organisms with genetically distinct cell types to study gene function in somatic tissues (Golic and Lindquist 1989). We used FLP/FRT mediated mitotic clonal analysis to elucidate the requirement for Spargel in salivary gland cells. Loss of GFP reporter in the salivary gland cells identifies Spargel-negative cells, and the Spargel signal (immunostained with Spargel-specific antibody) confirms the absence of Spargel (*srl^null^*) in specific salivary gland cells (Fig. 2a, a′, b, and b′). In contrast, the adjacent salivary gland cell with wild-type Spargel expression serves as a control (Fig. 2a, a′, b, and b′). *srl^null^* cells in the salivary gland cells have nuclei that are significantly smaller in size compared to the control cells (Fig. 2c and c′). Smaller nuclear size in the *srl^null^* salivary gland cell nuclei indicates that Spargel could be involved in endoreplication. Quantification of nuclear DNA content as measured through DAPI intensity of GFP-negative nuclei (Fig. 2d and d′) established that *srl^null^* clones carry half the DNA content compared to the adjacent control cells (Fig. 2e). Salivary gland cells are polyploid. The reduced DAPI intensity and smaller nuclear size in the *srl^null^* cell clones prompted us to investigate their endoreplication status.

**Fig. 3.**
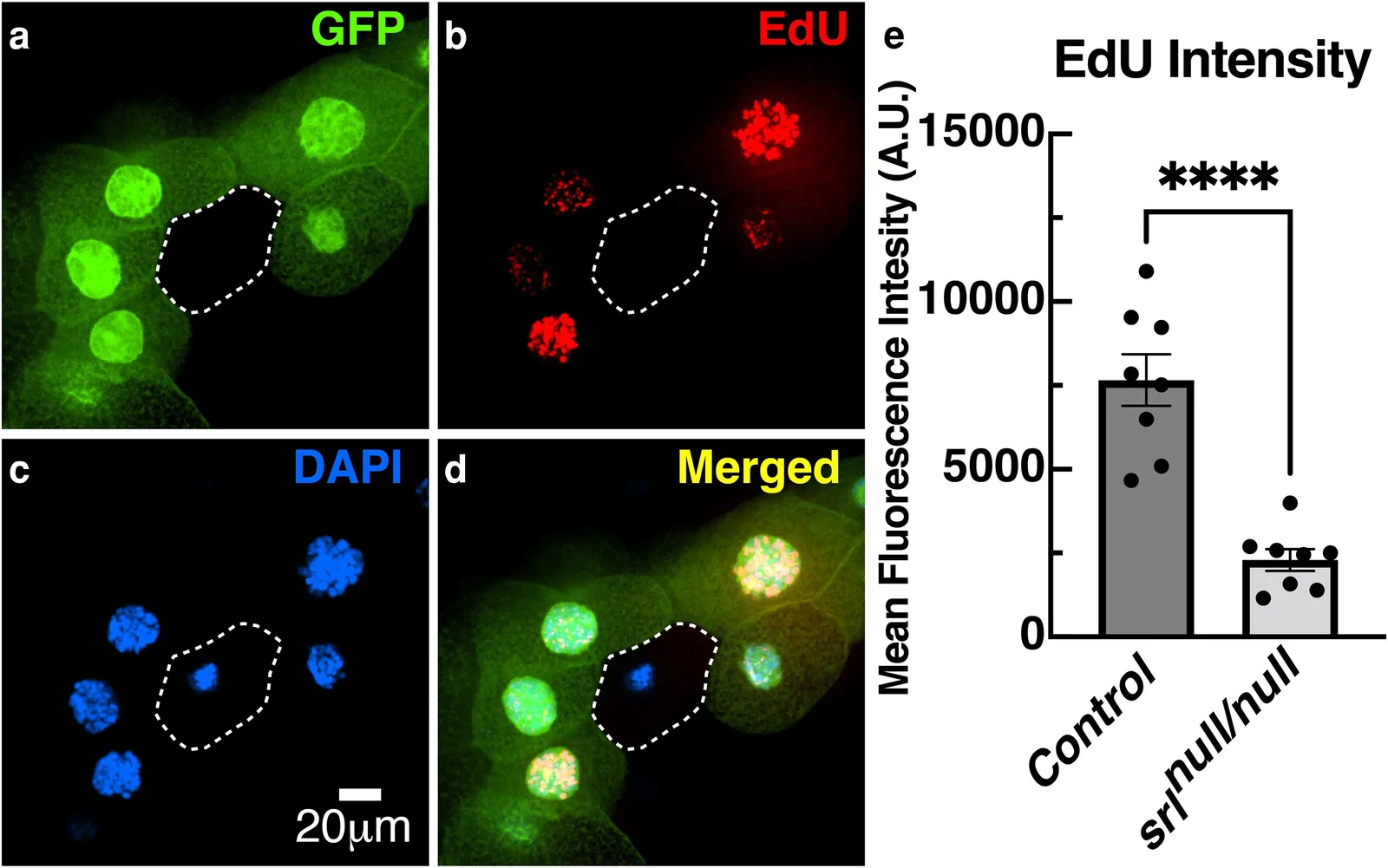
*Srl^null^* nuclei in the salivary gland undergo reduced endocycle. EdU incorporation into the clonal salivary gland to detect S-phase nuclei. a) GFP-negative cells in the salivary gland represent the *srl^null^* clone. b) Images of salivary glands following EdU incorporation support active EdU incorporation in *control* nuclei (GFP positive). However, *srl^null^* clone cells lack S-phase nuclei, as evidenced by no EdU incorporation (white-marked region). c) Representative images stained with DAPI show a smaller nucleus in the *srl^null^* clone, as indicated by the lack of GFP. d) Merged images. e) Comparison of EdU intensity between *control* cells and *srl^null^* clone cells. The number of dots represents the number of nuclei. The error bars represent SEM, and the *P* value is <0.0001. *n* = 8. White lines across all panels indicate the clone boundary. Scale bar 20 µm.

The endoreplication status of salivary gland cells was assessed by EdU incorporation, which labels S-phase nuclei (Buck et al. 2008). EdU incorporation is evident in control nuclei, indicating that at least one round of DNA replication has happened during the labeling period (Fig. 3a–d). In contrast, the absence of EdU signals in *srl^null^* cell clones validates the reduced endoreplication status of these cells (Fig. 3b). Quantifying the intensity of EdU-positive and -negative nuclei in both control and clonal groups (Fig. 3e) shows that the absence of Spargel almost completely halts DNA replication in the salivary gland.

### E2F1, a core regulator of endocycle, is nearly abolished in *srl^null^* cell clones

**Fig. 4.**
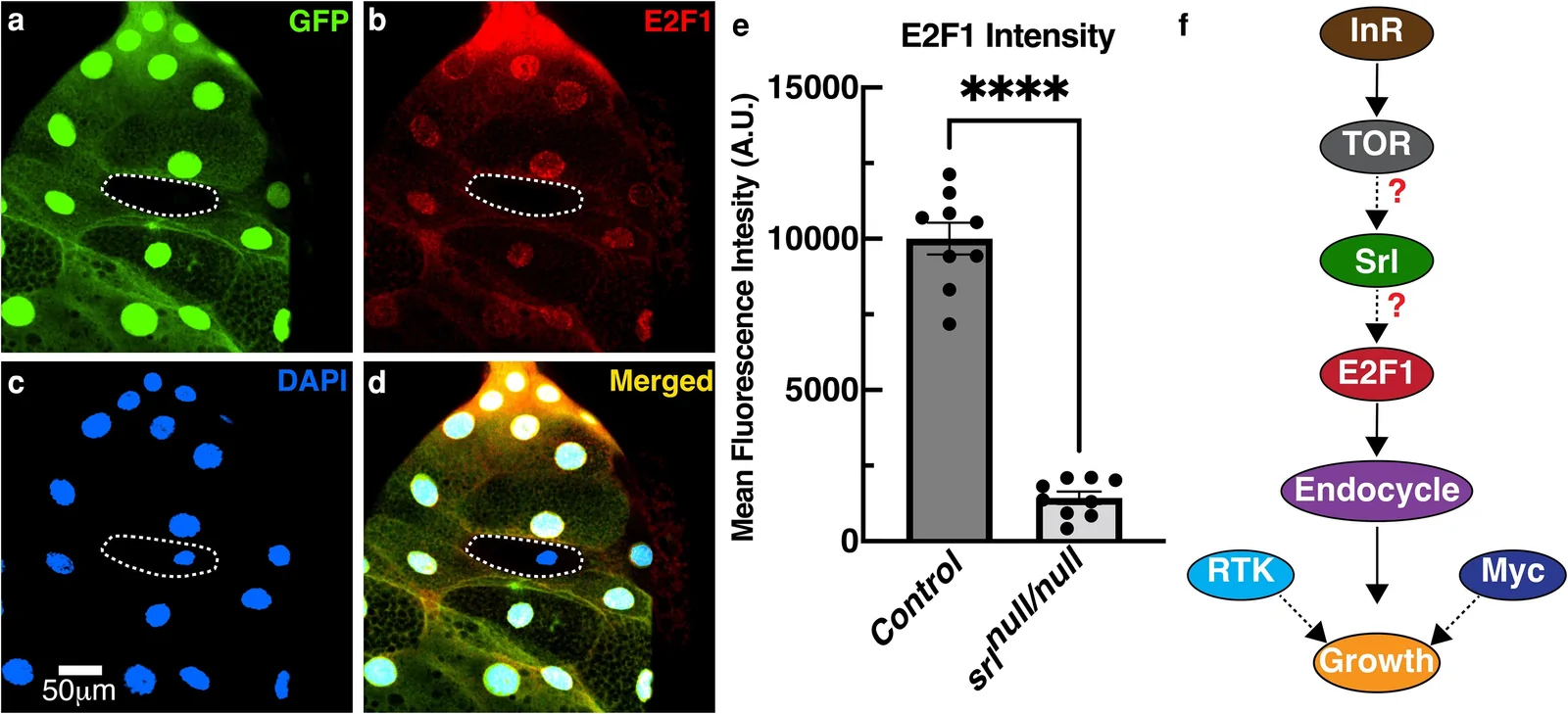
E2F1 expression is nearly abolished in *srl^null^* cells. a–d) Immunostaining with E2F1 antibody in the salivary gland containing the *srl^null^* clone. Absence of GFP expression, marked with a white dotted line, indicates the *srl^null^* clone a). E2F1 and DAPI-stained nuclei show a smaller nucleus in the *srl^null^* clone, as indicated by the lack of GFP expression in the nuclei of the *control* and the *srl^null^* clone (b and c, respectively) and merged image d). e) E2F1 intensity in the *control* cells compared to the *srl^null^* clone cells. The number of dots represents the number of nuclei. The error bars represent SEM, and the *P* value is <0.0001. *n* = 9. White dotted lines across all panels indicate the clone boundary. Scale bar 50 µm. f) The theoretical model incorporates Spargel within the endocycle-mediated pathway of the salivary gland, grounded on existing literature and the findings of the current study.

E2F1 is the core regulator of the endocycle in the *Drosophila* salivary gland, as its downregulation reduces endocycle (Zielke et al. 2013). Since cell-specific loss of function of *spargel* reduces DNA content by impairing DNA replication, this prompted us to profile E2F1 expression in the *srl^null^* cell clones. E2F1 expression is abrogated in *srl^null^* cell clones, while adjacent wild-type cells exhibit normal E2F1 expression (Fig. 4a–d). In addition, measurements of E2F1 intensity indicate that E2F1 proteins are nearly undetectable in the *srl^null^* cell clones (Fig. 4e). These data are quite significant, as we can now conclude that Spargel could be the upstream regulator of E2F1. Furthermore, Spargel and E2F1 work together to maintain proper endoreplication status and regulate cell growth. However, this study does not elucidate the comprehensive molecular mechanism by which Spargel regulates E2F1 expression and activity during this process. We propose a theoretical model of the endocycle pathway, positioning Spargel upstream of E2F1 based on current evidence and literature (Fig. 4f).

## Discussion

Endocycle is an alternative cell cycle program that allows cells to increase in size by repeatedly replicating their DNA content without undergoing cellular division (Edgar et al. 2014). The *Drosophila* larval salivary gland is a powerful model for elucidating how cellular- and tissue-level growth couples with the endocycle. The current study investigates the role of the PGC-1 ortholog Spargel/dPGC-1 in the endocycle process in the *Drosophila* larval salivary gland. Our findings identify Spargel as a crucial positive regulator of endocycle-mediated growth. Spargel modulates the expression of the core endocycle transcription factor E2F1 and acts upstream of E2F1. Using *Drosophila* genetics, we demonstrated that loss of Spargel in the larval salivary gland reduces nuclear size, accompanied by reduced EdU incorporation and near abolition of E2F1 expression. These findings position Spargel as an essential regulator that enables salivary gland cells to achieve normal ploidy and complete the endocycle, thereby supporting organ growth.

Endocycle is essential for determining adult body size in nematodes, which is also common in arthropods, including insects, and is well characterized in *Drosophila* (Zielke et al. 2013). Endocycle typically consists only of the S and G phases, resulting in polyploid cells with multiple copies of their genome (Shu et al. 2018). Skin, placenta, liver, and blood cells undergo endoreplication in mammals during development (Zielke et al. 2013). Endocycle and polyploidy are essential in many *Drosophila* larval tissues, including the salivary gland, gut, fat body, and adult tissues like ovarian nurse and follicle cells, gut, and glia for achieving normal sizes (Zielke et al. 2011; Fox and Duronio 2013; Zielke et al. 2013; Orr-Weaver 2015; Kim et al. 2021; Øvrebø et al. 2022).

The localization of Spargel in *Drosophila* is tissue specific. Spargel is expressed within the nuclei of germ cells (Basar et al. 2019; Jalal and Duttaroy 2024), and cytoplasmic expression of Spargel has also been documented in larval somatic cells (Tiefenböck et al. 2010; Mukherjee et al. 2013). The alternative subcellular localization of Spargel raises questions about the distribution of this protein between two distinct cellular compartments across different cell types and about the functional significance of this distribution. Under the tested conditions and imaging setup, we did not observe distinct nuclear Spargel expression in the salivary glands. However, this absence does not exclude potential nuclear localization. Thus, we cautiously propose that Spargel may function in the cytoplasm, a possibility that will require further experimental validation. One possibility is that cytoplasmic Spargel is regulated through post-translational modifications, such as phosphorylation. Consistent with this idea, Spargel has been identified as a candidate phosphoprotein in a phosphoproteomic analysis of *Drosophila* embryos (Zhai et al. 2008). However, in the salivary gland, we observed spatiotemporal changes in Spargel localization during development: Spargel was distributed more proximally in the gland during the third-instar larval stage, whereas in later stages, Spargel expression was observed throughout the gland. The salivary gland is a highly secretory and metabolically active tissue that undergoes dramatic growth and polyploidization. The expression of Spargel in the late-stage larval gland may provide mitochondrial and metabolic support, which could be crucial for fulfilling the elevated biosynthetic and energy demands associated with repeated endoreplication and secretion. Spargel expression in the later stages of the salivary gland could also contribute to histolysis of the gland.

Previous knockdown studies aimed at elucidating Spargel's function in somatic tissues and germ cells have demonstrated reduced growth (Mukherjee et al. 2013; Basar et al. 2019). Consistent with prior observations, a similar pattern of reduced growth has also been observed in both tissue-specific and global knockdown experiments. To further elucidate the role of Spargel in the larval salivary gland, we generated mitotic clones. The clonal analysis revealed that the complete loss of Spargel abolishes E2F1 expression. The *Drosophila* salivary gland is a unique tissue for studying the endocycle, in which the transcription factor E2F1 plays a central role in regulating this process (Kim et al. 2021). Salivary gland cells lacking *E2F1* block endoreplication, and E2F1 overexpression increases endoreplication, leading to hyperpolyploid cells (Zielke et al. 2011). Similarly, Spargel overexpression in the adult ovary results in larger ovaries with a greater number of mature eggs (Duttaroy lab, unpublished). In this observation, we demonstrated that E2F1 expression is Spargel dependent, meaning E2F1 functions downstream of Spargel (Fig. 4f). Since E2F1 plays a central role in the endocycle of nuclear DNA and ultimately in cellular and tissue growth, E2F1 being downstream of *spargel* easily explains the reduced cell, tissue, and eventually organismal growth resulting from the lack of Spargel protein. However, the detailed molecular mechanism by which Spargel regulates E2F1 remains unclear. DAPI staining and quantification demonstrated that, despite the absence of Spargel in the salivary gland, cells lacking Spargel continue to undergo the endocycle. This indicates that Spargel is not essential for initiating the endocycle, although it may affect its rate.

The TOR, Myc, and RTKs are the three upstream effectors that indirectly regulate the endoreplication pace in the *Drosophila* salivary gland (Edgar et al. 2014), as each affects DNA replication. These upstream factors also regulate the translation rate of *E2F1*, and alterations in these effectors lead to endocycle anomalies (Edgar et al. 2014). In a key observation, Tiefenböck et al. (2010) claimed that in fat body cells, growth is enhanced by overproduction of insulin receptor (InR), whereas the significant cell growth resulting from InR overexpression is abrogated in *spargel* hypomorph (*srl^1^*). Simultaneously, gene array analysis supported the induction of *srl* transcript levels in InR-overexpressing cells. In addition to InR, Spargel gain-of-function rescued cell-size and growth defects in other members of the insulin signaling pathway, such as TOR and S6K, in a cell-autonomous manner (Mukherjee et al. 2013). All this evidence points to the direct role of Spargel downstream of the Insulin-TOR signaling pathway (Tiefenböck et al. 2010; Mukherjee et al. 2013). Thus, like other members of the insulin signaling pathway, Spargel can also control cell growth. But the significant question that remains is how? Exclusion of E2F1 from *srl^null^* cell clones provides the strongest evidence that Spargel is involved in E2F1-mediated endoreplication and thus controls cell growth.

Future work will reveal: (1) how a transcription coactivator (Spargel) and transcription factor (E2F1) work together to control cellular growth and (2) how cytoplasmic Spargel can control the action of a transcription factor located in the nuclear compartment and regulate metabolism.

### Limitations of the study

This study presents several limitations that should be considered when interpreting the findings. First, our FLP/FRT-mediated clone design allows us to establish a clear requirement for Spargel in endocycle; however, it does not provide temporal resolution of individual endocycles. Consequently, we are unable to definitively determine whether Spargel primarily functions by slowing the overall pace of endocycling. Second, the EdU incorporation methodology restricts our capacity to directly correlate EdU intensity with the total number of completed endocycles, particularly in contexts of reduced ploidy. Third, we did not directly measure E2F1 transcript levels, E2F1 translational regulation, or Spargel-dependent transcriptional targets; therefore, we cannot yet ascertain whether Spargel primarily regulates E2F1 at the transcriptional, translational, or post-translational level. Future investigations involving stage-specific manipulations, time-resolved cell-cycle reporters, and metabolic measurements will be essential to address these limitations and to more accurately elucidate how Spargel coordinates growth, metabolism, and E2F1-mediated endocycle regulation.

## Data Availability

All data reported in this article that support the findings of the study are publicly accessible as of the publication date. Imaging, quantitative data, and any other numerical datasets used to produce the graphs can be obtained from the corresponding author upon request. Fly strains are available upon request to the corresponding author.
